# Dietary inflammatory index and its relation to the pathophysiological aspects of obesity: a narrative review

**DOI:** 10.20945/2359-3997000000631

**Published:** 2023-06-19

**Authors:** Roseli Neves de Mello, Bárbara Paixão de Gois, Ana Claudia Pelissari Kravchychyn, Ana Raimunda Dâmaso, Maria Aderuza Horst, Glaucia Carielo Lima, Flávia Campos Corgosinho

**Affiliations:** 1 Universidade Federal de Goiás Faculdade de Nutrição Programa de Pós-graduação em Nutrição e Saúde Goiânia GO Brasil Universidade Federal de Goiás, Faculdade de Nutrição, Programa de Pós-graduação em Nutrição e Saúde, Goiânia, GO, Brasil; 2 Universidade Federal de Viçosa Programa de Pós-Graduação em Ciência da Nutrição Viçosa MG Brasil Universidade Federal de Viçosa, Programa de Pós-Graduação em Ciência da Nutrição, Viçosa, MG, Brasil; 3 Universidade Federal de São Paulo Escola Paulista de Medicina Programa de Pós-graduação em Nutrição São Paulo SP Brasil Universidade Federal de São Paulo, Escola Paulista de Medicina, Programa de Pós-graduação em Nutrição, São Paulo, SP, Brasil; 4 Universidade Federal de Goiás Programa de Pós-graduação e Ciências da Saúde Goiânia GO Brasil Universidade Federal de Goiás, Programa de Pós-graduação e Ciências da Saúde, Goiânia, GO, Brasil

**Keywords:** Inflammation, biomarkers, diet, weight gain, cytokines

## Abstract

Obesity, a complex disease that involves energy imbalance and chronic low-grade inflammation, is implicated in the pathogenesis of several chronic non-communicable diseases. As dietary components modulate the human body's inflammatory status, the Dietary Inflammatory Index (DII^®^), a literature-derived dietary index, was developed in 2009 to characterize the inflammatory potential of a habitual diet. Abundant research has been conducted to investigate the associations between DII and obesity. In this narrative review, we examined the current state of the science regarding the relationships between DII and the inflammatory pathophysiological aspects related to obesity. DII is associated with inflammation in obesity. The most pro-inflammatory diet was directly related to higher levels of pro-inflammatory markers, which included C-reactive protein (CRP), interleukin-6 (IL-6), IL-1β, and tumor necrosis factor-α (TNF-α). Therefore, evidence suggests that the use of the DII may be useful for understanding the relationship between diet and the inflammatory process related to obesity.

## INTRODUCTION

Obesity is characterized by the excessive accumulation of body fat and potential health impairments. In 2016, 650 million adults worldwide were diagnosed with obesity, representing 13% of the adult population (11% for men and 15% for women) (1). Obesity is also associated with a high risk of comorbidities such as cardiometabolic disease, non-alcoholic fatty liver disease, metabolic syndrome, type 2 diabetes, hypertension, dyslipidemia, coronary artery disease, congestive heart failure, obstructive sleep apnea, depression, anxiety, arthritis, and several types of cancers (2-6). Moreover, in 2015, obesity-related diseases were responsible for more than 2.4 million adult deaths worldwide (2).

Obesity is a multicomponent disease caused by the prolonged maintenance of positive energy balance (7). Excess energy is stored as triglycerides in adipocytes mainly through cell hypertrophy. Due to impaired angiogenesis, local hypoxia occurs, leading to cellular organelle failure and a consequent increase in oxidative stress. This state activates toll-like receptors (TLR) and the nuclear factor (NF)-kB pathway, giving rise to an inflammasome, a multi-protein complex that is responsible for the release of several inflammatory cytokines. Finally, macrophage infiltration occurs to phagocytize adipocyte dendrites, resulting in a low-grade inflammatory state (8). This low-grade inflammation causes liver dysfunction, resulting in increased expression of inflammatory markers such as C-reactive protein (CRP), serum amyloid A, and fibrinogen; in the muscle cells, it causes increased expression of IL-6, TNF-*α*, and myokines (9). Thus, chronic inflammation has been proposed to be a link between obesity and the development of cardiometabolic diseases (10).

Among the many factors responsible for unbalanced energy, low energy expenditure, physical inactivity, and overeating play major roles. Overeating frequently includes the consumption of high-calorie, low-quality food. Foods and beverages contain components that can act on inflammatory pathways in the peripheral tissues, changing the plasma concentration of inflammatory markers and influencing the health of individuals (11). Therefore, researchers have developed the dietary inflammatory index (DII)^®^, which can assess the dietary quality in relation to its inflammatory potential and, consequently, the risk of disease (12). In several studies, including systematic reviews and meta-analyses, high DII^®^ scores were found to be associated with cardiovascular diseases (13,14), cancer (15-17), metabolic syndrome (18,19), and obesity (20,21). From this perspective, the present literature review aimed to describe the DII^®^ and discuss its relationship with the pathophysiological aspects of the inflammatory process associated with obesity and cardiometabolic diseases. Therefore, we will first introduce the concept of obesity, its etiology, physiopathology, and how food can modulate this process. Afterward, the DII^®^ and its correlation with obesity and cardiometabolic status will be described.

## ETIOLOGICAL AND PATHOPHYSIOLOGICAL ASPECTS OF OBESITY

The etiology of obesity is complex and involves the interaction between biological, environmental, and behavioral factors (7,22,23). Weight gain occurs when energy intake exceeds energy expenditure, favoring a positive energy balance (7,22,23). When this energy balance is maintained for a prolonged period, the excess energy is stored in the adipocytes, promoting adipocyte tissue expansion and obesity (10). Obesity can lead to adipose tissue dysfunction by promoting immune cell infiltration, impaired angiogenesis, local hypoxia, fibrosis, and unregulated production of adipokines (24,25). Adipokines are molecules (hormones, cytokines, chemokines, and other proteins) secreted by the adipose tissue, which have the function of regulating metabolic homeostasis and immune reactions, among other local and systemic functions (8,26-31).

These changes lead to a state of chronic low-grade inflammation that contributes to the development of comorbidities (8,10,26,32,33). Hypertrophied adipocytes undergo structural alterations and are subjected to cytotoxic stress. These events may result in pyroptosis and death of adipocytes by disruption of the plasma membrane, which subsequently results in the release of pro-inflammatory cellular content into the extracellular environment (34).

Free lipid droplets of cholesterol and cytotoxic fatty acids are released by dead adipocytes and need to be eliminated to avoid damage to adipose tissue cells (35). Macrophages infiltrate the adipose tissue to clear the extracellular space of the adipocyte debris (35). The number of macrophages in the adipose tissue is higher in individuals with obesity than in lean individuals. Under these conditions, more than 90% of the macrophages are located around dead adipocytes, forming crown-like structures (CLS) (35). Finally, the macrophages fuse to form multinucleated giant cells (MGCs), a marker of chronic inflammation (35).

Additionally, obesity induces a phenotypic change in the macrophages from an anti-inflammatory M2 polarization state to a pro-inflammatory M1 state (27). Activated M1 macrophages secrete pro-inflammatory cytokines that play important roles in chronic inflammation in obesity, such as interleukin (IL)-6, IL-1β, and monocyte chemoattractant protein (MCP)-1 (25,27,35). Additionally, they release tumor necrosis factor-*α* (TNF-*α*), nitric oxide (NO), and reactive oxygen species (ROS), which can complement and amplify the mechanisms of adipocyte death (35). Thus, macrophage infiltration in the adipose tissue is implicated in inflammation and metabolic complications of obesity (35).

**Figure 1:**
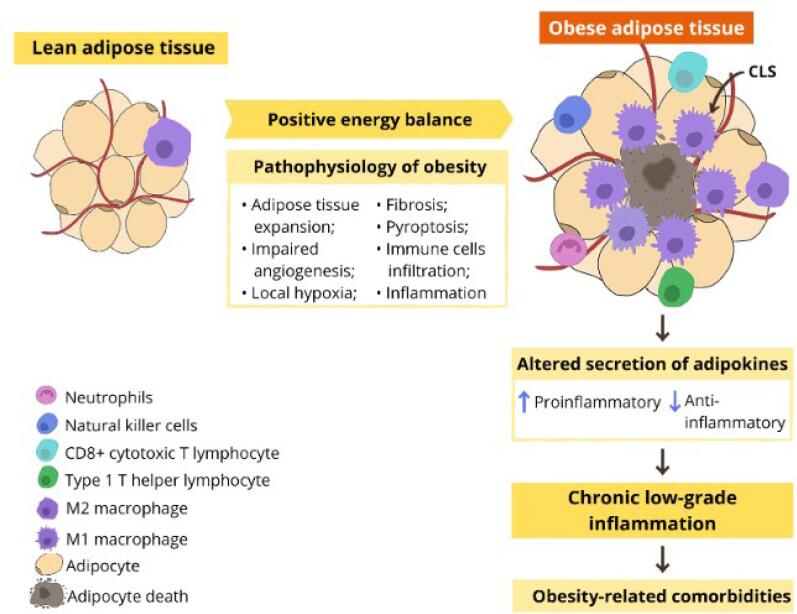
Obesity: etiological and pathophysiological aspects and the inflammation of adipose tissue and its effects. In obesity, there is a greater infiltration of immune cells in the adipose tissue, a change in phenotype from anti-inflammatory (M2) to pro-inflammatory (M1) macrophages, and dysregulated secretion of adipokines. Macrophages surround the adipocytes that have undergone pyroptosis and form crown-like structures (CLS), whose purpose is to remove debris from these adipocytes. Blue arrows indicate increased secretion (when pointed up) or decreased secretion (pointed down). Adapted from Calder el al. (48).

Furthermore, the levels of other innate and adaptive pro-inflammatory immune cells also increase in the adipose tissue in patients with obesity, such as natural killer cells, neutrophils, type 1 helper T lymphocytes, and cytotoxic CD8+ T lymphocytes (27,28). These cells also contribute to inflammation by producing pro-inflammatory factors, such as elastase, interferon-gamma (IFN-*γ*), myeloperoxidase (MPO), and TNF (36). Figure 1 illustrates the main adipose tissue changes in a state of a chronic positive energy balance.

In addition to storing energy, the adipose tissue has other functions, especially endocrine functions, such as secretion of adipokines (29). Adipokines regulate metabolic homeostasis and immune reactions, with local or systemic action (8,26-31), and their secretion is altered in the presence of obesity. In individuals with obesity, the production of pro-inflammatory adipokines (leptin, TNF-*α*, resistin, visfatin, IL-6, and IL-1β) is increased and that of anti-inflammatory cytokines (*e.g.*, adiponectin) is decreased (24,25).

Adipokines regulate the secretion of acute-phase proteins in other organs, such as the liver, and in the case of low-grade systemic inflammation, there is an increase in the production of CRP, amino acids (AAs), fibrinogen, and haptoglobin (37). Of note, as the degree of obesity increases, the levels of inflammatory markers being secreted by the adipose tissue also increase. In fact, a cross-sectional study of 10,745 adults and older individuals reported increased levels of inflammatory markers (white blood cell count, platelet count, erythrocyte sedimentation rate, and CRP) as the BMI increased (p < 0.0001) (38).

## IS INFLAMMATION A PRECURSOR OF OBESITY?

Although inflammation is considered a consequence of obesity or weight gain, some studies have suggested a bidirectional relationship in which inflammation could play an etiological role (39-42). From this perspective, Holz and cols. (40) pointed out that individuals with high concentrations of inflammatory markers are more likely to gain weight. In their study, among the 2,792 middle-aged adults followed for approximately 10 years, as compared with individuals in the lowest quartiles, those in the highest quartiles of fibrinogen, CRP, and WBC count were likely to experience high average annual weight gain (mean, 1.02 kg/year), at odds ratios of 1.73 (CI 95%, 1.27-2.35), 1.45 (CI 95%, 1.08-1.94), and 1.37 (CI 95%, 1.03-1.82), respectively.

Corroborating these findings, Duncan and cols. (41) found a 65% increase in the relative risk of severe weight gain (greater than the 90^th^ percentile) in individuals in the highest fibrinogen quartile compared with those in the lowest. In this cohort, which followed 13,017 adults aged 45 to 64 years for over 3 years, an increase of 0.23 kg/year was observed when individuals at the extremes of fibrinogen quartiles were compared. In addition, elevated levels of inflammation-sensitive plasma proteins (fibrinogen, *α*1-acid glycoprotein, *α*1-antitrypsin, haptoglobin, and ceruloplasmin) have also been found to predict weight gain (42).

Therefore, although it is still unclear whether increased levels of inflammatory markers precede weight gain, anti-inflammatory interventions targeted mainly at individuals with elevated concentrations of inflammatory markers could be an important approach for preventing obesity. Many factors might affect the onset of inflammation, including age, level of physical exercise, sex, genetics, tobacco use, gut microbiota, drug use, stress levels, sleep quality, pollution exposure, viral infection, and diet (9). Dietary aspects have recently gained attention due to their pro- and anti-inflammatory properties and are the focus of this review (43).

## DIETARY CAPACITY TO MODULATE THE INFLAMMATORY PROCESS

Besides providing essential nutrients for humans, food contains non-nutrient bioactive compounds that have therapeutic potential and may influence energy intake, while reducing the pro-inflammatory response, oxidative stress, and risk of metabolic disorders. Although these dietary components are not limited to plants, the benefits of plant-derived compounds (phytochemicals) have been highlighted. In fact, the consumption of fish products can provide components, such as n-3 fatty acids, which may also influence inflammatory pathways. Table 1 summarizes the information on bioactive compounds in the context of inflammation (44,45).

**Table 1:** A synthesis of food bioactive compounds and their main transcription factors targets

Food Bioactive Compound	Food source	Anti-Inflammatory transcription factors target
6-Shogaol	Ginger	Activation of PPAR_γ_ and Modulation of JNK signaling pathways
Allicin	Garlic	Activation of Nrf2 and Inactivation of NF-κB
Capsaicin	Red pepper	Inactivation of NF-κB
Catechins	Green tea	Activation of Nrf2 and Inactivation of NF-κB
Cinnamaldehyde	Essential oil from bark and cinnamon	Activation of MAPK pathways and activation of Nrf2
Curcumin	Turmeric	Activation of Nrf2
Diosgenin	Fenugreek, yam roots	Inactivation of NF-κB and Modulation of JNK signaling pathways
Isohumulone	Humulus lupulus hop	Agonist of PPAR_γ_
Luteolin	Herb, Spice	Modulation of JNK signaling pathways
Lycopene	Tomato, watermelon	Activation of MAPKs and Nrf2, Inactivation of NF-κB and AP-1
Naringenin, Auraptene	Citric fruits	Modulation of JNK signaling pathways and agonist of PPAR_γ_
PUFA (EPA and DHA)	Fish oil	Inactivation of NF-κB by PPAR_γ_ activation
Resveratrol	Grapes and red wine	Activation of Nrf2
Sulforaphane	Cruciferous vegetables	Activation of Nrf2 and Inactivation of NF-κB

Abbreviations: AP-1, Activator protein-1; JNK, c-Jun N-terminal kinase; MAPK, mitogen-activated protein kinase; NF-κB, nuclear factor kappa B; Nrf2, nuclear factor E2-related factor 2; PPAR_γ_, Peroxisome proliferator activated receptor gamma. Data extracted from the references 45 and 46.

Phytochemicals reduce inflammation via kinases such as protein kinase C (PKC), c-Jun kinase (JNK), and mitogen-activated protein kinase (MAPK). Phytochemicals inhibit these enzymes by altering the DNA-binding capacity of transcription factors such as nuclear factor kappa-B (NF-kB). Consequently, the expression rates of the target genes are controlled. NF-kB is a major effector pathway that is involved in inflammation. Phytochemicals also exert anti-inflammatory action through the activation of the nuclear factor E2-related factor 2/antioxidant response element (Nrf2/ARE) pathway, which is the most important pathway that protects cells against oxidative stress, consequently reducing inflammation (44-46).

**Figure 2:**
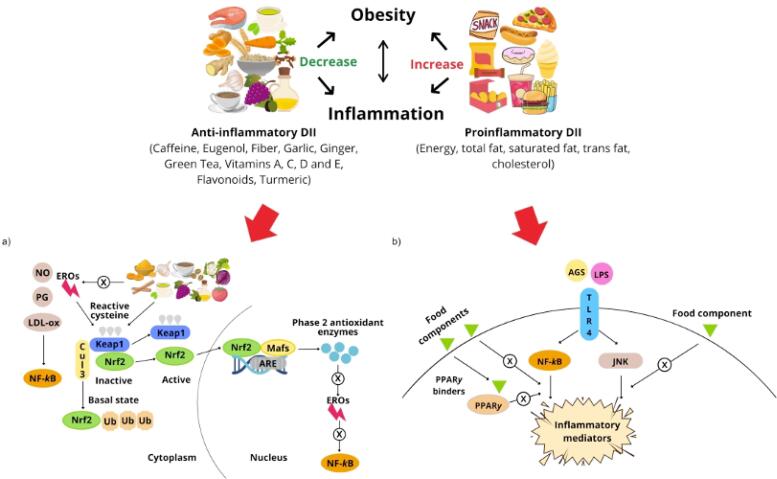
Relationship between the dietary inflammatory index, inflammation, and obesity. A negative dietary inflammatory index (anti-inflammatory DII) is associated with reduced inflammation and lower body weight, while a positive dietary inflammatory index (pro-inflammatory DII) is associated with increased inflammation and obesity. It is known that obesity increases blood inflammatory markers. However, some studies have shown a bidirectional action, where higher inflammatory markers could lead to obesity. a) Modulation of inflammation by food components through erythroid nuclear factor 2 related to factor 2 (Nrf2): Nrf2 is a transcription factor that is activated in response to oxidative stress and substances such as nitric oxide (NO), oxidized low-density lipoproteins (LDLox) and prostaglandins (PG). Nrf2, after activation, translocates to the nucleus, binds to Maf proteins (musculoaponeurotic fibrosarcoma) and to the antioxidant response element (ARE). In this way, it regulates genes that express enzymes with antioxidant and anti-inflammatory actions. Bioactive compounds can activate Nrf2, possibly by altering reactive cysteines within the Kelch-like ECH-associated protein 1 (Keap1), an adapter component of the Cul3 complex. As Nrf2 limits the levels of reactive oxygen species (ROS), food compounds can also lead to inhibition of nuclear factor-kB (NF-kB), as it affects its signaling pathway, involved in inflammation. b) Signaling pathways of inflammatory gene expressions and dietary strategies capable of modulating inflammation: Saturated fatty acids (AGS) and lipopolysaccharides (LPS) bind to toll-like receptor 4 (TLR4), which induces inflammatory responses via nuclear factor-κB (NF-κB) and c-Jun N-terminal kinase pathways (JNK), important modulators of inflammatory gene expression. On the contrary, some food components can act by directly inhibiting these pathways. The peroxisome proliferator-activated receptor γ (PPARγ) acts by attenuating inflammation. Thus, PPARγ-binding food components may help to improve the inflammatory response.

Finally, some of the bioactive compounds in food act as agonists of peroxisome proliferator-activated receptor gamma (PPAR*γ*) and mediate the resolution of inflammation by increasing the levels of adiponectin, resolvin, and protectin as well as by inducing protein G-coupled receptor (GPR120)-mediated inhibition of the NF-*κ*B pathway (Figure 2) (44,47).

However, diet can also provide components that have pro-inflammatory effects, such as saturated fatty acids (SFA), oxidized lipids, glucose, and fructose (48). These components can activate TLRs and NF-*κ*B-associated pathways, which have been proposed as the primary molecular mechanisms mediating adipose tissue inflammation by inducing the expression of pro-inflammatory genes (44).

Considering that food components can interact with each other in a synergistic or antagonistic manner, many studies have evaluated food patterns instead of isolated components. The Mediterranean diet, which is rich in fruits, vegetables, nuts, whole grains, olive oil, and fish, is associated with reduced serum concentrations of inflammatory markers (49,50). In contrast, diets rich in refined foods, sugar, red and processed meats, and fried foods increase the levels of these inflammatory markers (51).

Overall diet quality can be measured using different indicators, generally based on nutrient intake, number of servings consumed, and diet variety. Most dietary indices are based on measures of nutrient intake, food or food groups, or both (52). In this context, the DII^®^ was developed for evaluating dietary quality in relation to its inflammatory potential that could influence the risk of diseases, considering many dietary components and not just one isolated food item/nutrient/bioactive compound (12).

## DIETARY INFLAMMATORY INDEX

### History, development, and calculation of the DII

The DII^®^ was originally developed in 2009 after an extensive literature review of articles published between 1950 and 2007 that evaluated the combined effect of dietary parameters (whole foods, nutrients, and dietary constituents) on specific inflammatory markers [IL- 1β, IL-4, IL-6, IL-10, tumor necrosis factor [TNF]-*α*, and CRP]. The first version of the DII^®^ was based on 927 peer-reviewed articles published in English in the biomedical literature (12). The authors evaluated the validity of DII^®^ through its effect on high-sensitivity CRP (hs-CRP), using data from the longitudinal observational Seasonal Variation of Blood Cholesterol Study (SEASONS). The results showed no significant association when hs-CRP level was examined as a continuous variable. However, using another strategy, by dichotomizing the hs-CRP values into higher and lower than 3 mg/L, the DII^®^ showed a significant and inverse association with hs-CRP (β = −0.06; p = 0.049). This result may reflect the nonlinearity of the relationship between hs-CRP and diet (12).

In 2013, van Woudenbergh and cols. (53) proposed the adapted DII^®^ (ADII) – this version was adjusted for energy and fat intake, variation in the consumption of dietary components, and inclusion of ethanol. In 2014, the index was further improved by extending the literature review to articles published up to 2010, expanding the list of dietary parameters to 45 items, and refining the scoring algorithm by building a global food consumption database that serves as a reference for measurement (43). As overall dietary energy intake was strongly associated with DII scores in some populations, the authors created an energy-adjusted DII (E-DII). The use of the E-DII improved prediction compared to unadjusted DII scores (54). Furthermore, another version was developed to include the children's population, called the child's DII^®^ (C-DII). The C-DII was validated with data from children aged 6-14 years (n = 3.300) and showed an association between CRP concentrations and C-DII scores (55).

The pro-and anti-inflammatory fractions of each food parameter were calculated in the DII^®^ by dividing the weighted results of the articles that showed pro-and anti-inflammatory effects by the total weighted number of articles. The anti-inflammatory fraction was subtracted from the pro-inflammatory fraction to calculate the raw score for the inflammatory effect of each food parameter. To account for the robustness of the literature, when the total weighted number of articles on a particular food parameter was less than 236 (median of the total weighted number of articles on all food parameters), this value was divided by 236 and multiplied by the raw score of the inflammatory effect (43). The following parameters were classified as pro-inflammatory: calories, carbohydrates, proteins, total fat, saturated fat, trans fat, cholesterol, iron, and vitamin B12. In contrast, monounsaturated and polyunsaturated fats, omega-3, omega-6, fiber, beta-carotene, folic acid, thiamine, riboflavin, niacin, pyridoxine, retinol, ascorbic acid, tocopherol, vitamin D, magnesium, selenium, zinc, alcohol, caffeine, clove oil, garlic, ginger, onion, turmeric, saffron, green and black tea, flavones, flavonols, flavanones, isoflavones, anthocyanidins, pepper, thyme/oregano, and rosemary were classified as anti-inflammatory (43).

The current DII^®^ is connected to a global food intake database to relate individual intake to this global pattern to avoid arbitrary arithmetic manipulations and to regulate the influence of each food parameter on the final DII value. Individual intakes of each dietary parameter are transformed into z-scores and centered percentiles, based on the universal mean and standard deviation. Z-scores and percentiles are the same regardless of the units of measurement, canceling out their effect on the results. The centered percentile value is multiplied by the overall dietary parameter effect score to obtain the DII score of the parameter in question. The DII of each food parameter is summed to obtain an individual's overall DII score. The authors provided seven scenarios of DII scores obtained from a variety of plausible intakes of the 45 food parameters which ranged from +7.98 (maximum pro-inflammatory) to −8.87 (maximum anti-inflammatory) (43).

Similar to the initial DII, the current DII was validated using SEASON data, and the hs-CRP was evaluated as a dichotomous dependent variable (≤3 mg/L, >3 mg/L) (56). However, food consumption data for the current DII were collected through 24-hour food recalls and structured food consumption questionnaires for the last 7 days. The DII calculated from both recalls (24 h and 7 days) was associated with high hs-CRP levels. A 5-point increase in the DII score derived from the 24-h recall was associated with an almost 50% increase in the odds of elevated hs-CRP (OR, 1.47; 95% CI, 1.03-2.10). Likewise, a 5-point increase in the score derived from the 7-day recall was associated with a 60% increase in the odds of elevated hs-CRP (OR, 1.60; 95% CI, 1.09-2.36). This new population-based DII was found to be more strongly correlated with CRP-us (r = 0.11, P < 0.0001) than the initial DII (r = 0.04, P = 0.08) (57).

The 24-hour recalls are more suitable for measuring the consumption of spices and other parameters that may have an effect on inflammation. Structured questionnaires present a limited representation of dietary information and therefore fail to measure some parameters such as spices (57). In the validation of the DII, 28 dietary parameters were used for the calculation of the DII in the structured questionnaire, while in the 24-hour recall, 44 parameters were used. Even so, both have similar abilities for predicting elevated hs-CRP levels. However, the authors argue that the similarity in the responses to the structured questionnaire and 24-h recall may be due to the low consumption of these foods in the population studied (57). Thus, the use of structured questionnaires may be a limitation of the tool since they do not include important food components, especially in populations that have a dietary pattern rich in spices and other bioactive compounds.

### DII and inflammatory biomarkers

Diet is a possible mechanism for modulating inflammation. The DII is a useful tool for measuring the potential impact of diet on the inflammatory status of an individual. As mentioned previously, this index has been validated using data from the SEASONS study. An association between the DII score and elevated hs-CRP (>3 mg) was found in groups using 24-hour dietary recall (OR, 1.08) and 7-day food recall (OR, 1.10) (57).

Other studies have confirmed a direct relationship between the DII and the concentration of pro-inflammatory cytokines. In a study involving 2,487 Belgian individuals (aged 35 to 55 years), for each unit increase in the DII, the risk of having IL-6 >1.6 pg/mL and homocysteine >15 μmol/L increased by 19% and 56%, respectively. However, no significant associations were found between the DII and hs-CRP (>3 mg/L) or fibrinogen (>4.5 g/L) (56).

Kotemori and cols. (58) found a positive association between the DII and inflammation status in Japanese men; participants in the upper quartile of the E-DII were 72% more likely to have elevated hs-CRP concentrations (>3 mg/L) than those in the lower quartile (OR: 1.72). In contrast, no association was observed between E-DII score and hs-CRP concentration in women, except in those who did not take prescription drugs. One justification for the discordant findings between the sexes is that the diets of women in this study had a significantly lower inflammatory potential than those of men, where women had a negative mean E-DII score, whereas men had a positive mean E-DII score (58). Thus, further studies are needed to better investigate the applicability of this tool in associating diets with inflammatory markers in different populations.

### DII and obesity

Considering that dietary inflammatory properties may play a role in regulating adipose tissue inflammation (11), studies have been conducted to verify the association between the DII score and risk of obesity. Among 7,027 healthy undergraduates of the *Seguimiento Universidad de Navarra* (SUN) cohort, the highest quartile of DII was associated with a higher average annual weight gain and higher risk of being overweight and developing obesity than that associated with the lowest quartile (20). Corroborating these findings, a cross-sectional study of 3,151 undergraduate and graduate students from two Brazilian public universities conducted by Oliveira and cols. showed an association between a pro-inflammatory DII and a high prevalence of overweight and obesity (59).

From the perspective of weight loss interventions using DII analysis, Muhammad and cols. (60) subjected 57 overweight and adults with obesity to a dietary intervention program. The subjects were instructed to follow either of the two interventions: a) a low-calorie diet of 1,250 kcal/day for 12 weeks or b) a very low-calorie diet of 500 kcal/day for 5 weeks. The two strategies were aimed at 10% reduction in body weight. After this stage, they went through a weight maintenance period that consisted of a stable weight period (balanced diet, based on individual energy needs and according to the Dutch national dietary guidelines for 4 weeks) and a follow-up period (advised to maintain body weight, received no further dietary consultations, with the freedom to choose their diet for 9 months). After the follow-up period, the weight change varied from −3.8 kg to +13.5 kg. However, a positive correlation was found between the DII during the maintenance phase and weight gain (r = 0.287, p = 0.045). It is important to note that the authors did not group the volunteers according to BMI (overweight and obesity); thus, it was not possible to observe differences in the results between overweight and obesity conditions (60).

Kord Varkaneh and cols. (61) performed a meta-analysis based on articles published until 2017, which included 22 studies on the effect of the DII on BMI and four studies that included data on the hazard risk (HR) or ORs for obesity. A significant difference in BMI (mean difference, 0.811) was found between the highest and lowest DII categories. An association was also found between the DII, which compared the groups with the highest and lowest BMIs, and obesity (OR = 1.310); it was concluded that high DII categories were associated with increased BMI and obesity.

In addition, Farhangi and Vajdi (21) investigated the association between the DII and central obesity rates in the general population through a systematic review and meta-analysis of 32 observational studies. The findings showed that adherence to diets with higher DII scores was associated with a 1.81 cm increase in waist circumference (pooled weighted mean difference (WMD) = 1.813; CI, 0.785-2.841; p = 0.001). The sources of heterogeneity across studies were continent, dietary assessment tools, and sex.

An inflammatory diet may play a role in the development of overweight or obesity in both children and adolescents. Aslani and cols. (62) observed that children and adolescents (n = 5,427) in the last quartile of the DII had higher BMI z-scores and higher waist, neck, and hip circumferences than those in the first quartile (trend p < 0.001, for all comparisons). After multiple linear regression analysis, with adjustments for potential confounders, students in the highest DII quartile showed higher BMI z-scores and waist and hip circumferences than those in the lowest quartile (p < 0.05 for all comparisons) (62).

One of the suggested mechanisms by which a pro-inflammatory DII could favor obesity and weight gain is through increased levels of circulating inflammatory markers. However, studies evaluating the association between DII scores and risk of obesity have presented contradictory results. In contrast to the above results, in their study conducted in Myanmar, San and cols. (63) found no significant difference between the overall mean DII scores of 244 female teachers with and without obesity. Camargo-Ramos and cols. (13) conducted a cohort study of 90 Colombian overweight adults (BMI ≥ 26 and ≤ 35 kg/m^2^) who were sedentary and had at least one criterion for metabolic syndrome. The samples were divided into two groups: pro-inflammatory (positive values) and anti-inflammatory (negative values). Anthropometric measurements (weight, BMI, waist circumference, and hip circumference) and body composition parameters (total tissue, fat, and lean mass in grams) and percentages (trunk, gynecoid, android, android/gynecoid, and fat quotient) were measured by dual-energy X-ray absorptiometry (DXA). No differences were found between the groups in the frequencies of overweight and obesity and anthropometric and body composition variables. However, the absence of significant associations may be explained by the homogeneity of the sample in terms of anthropometric characteristics (13).

In a cross-sectional study conducted with 503 Indonesian adults, Muhammad and cols. (11) reported that the DII score was not associated with any of the anthropometric measures evaluated (body weight, BMI, body fat, waist circumference, and hip circumference), systolic and diastolic blood pressure, or lipid profile (triglycerides and high-density lipoprotein [HDL-c]). However, the authors suggested that this lack of association may be a consequence of the sample being composed only of healthy people, unlike the general population in Indonesia.

### DII and cardiometabolic diseases

Several studies have investigated the association between diet-generated inflammation, evaluated using the DII, and the risk of cardiometabolic diseases. A cross-sectional study of 366 Croatian workers with sedentary occupations showed that individuals with a high DII were twice as likely to have metabolic syndrome and a pro-inflammatory diet was associated with central obesity, hypertriglyceridemia, hypertension, and low-density lipoprotein (18).

Another study with 9,291 Korean adults found a positive association between the DII and the prevalence of hyperglycemia in men and central obesity in postmenopausal women, but not in premenopausal women (19). A population-based cross-sectional probabilistic survey found that Mexican adults (n = 1,174) with a more pro-inflammatory diet were three times more likely to have type 2 diabetes than individuals in the lowest DII quintile. Furthermore, the magnitude of this association is pronounced among individuals with overweight/obesity, low levels of physical activity, and advanced age (64).

Moreover, increased DII scores are associated with increased all-cause mortality and cardiovascular disease in individuals with a metabolically unhealthy obesity phenotype, as reported in a nationally representative study of US adults that analyzed the data of 3,733 adults who presented overweight or obesity (from the National Health and Nutrition Examination Survey III, 1988-1994) (65). In contrast, a prospective cohort study of 6,016 American men found no evidence that a pro-inflammatory diet was associated with the incidence of type 2 diabetes mellitus after 6.5 years of follow-up (66). Taken together, these studies show the important role of the DII, suggesting that it might be a useful tool in clinical practice for screening patients with metabolic disorders using diet information.

Considering the youth population, a study performed with 31,684 Brazilian adolescents it was found a positive association between a high pro-inflammatory diet with high HOMA-IR among boys; while among girls high pro-inflammatory diet was associated with high fasting glucose, LDL-cholesterol, triglycerides, and low HDL-cholesterol. Furthermore, when analyzed a moderately pro-inflammatory diet, it was found an association with high HOMA-IR in the girls’ group and high total cholesterol among boys (67). Finally, a clinical trial with 45 adolescents enrolled for long-term interdisciplinary therapy, including clinical, nutritional, psychological counseling, and exercise training, showed that a decrease in DII scores was associated with a decrease in the cardiometabolic risk, suggesting that an anti-inflammatory diet may be a good strategy for treating and controlling obesity and related comorbidities (68).

In addition, Ji and cols. (14) performed a systematic review and meta-analysis of 15 cohort studies whose objective was to evaluate the effect of DII on the incidence of and mortality from cardiovascular disease (CVD). They observed a significant association between high DII scores and high risk of CVD incidence (RR, 1.41; 95% CI, 1.12-1.78) and mortality from CVD (RR, 1.31; 95% CI, 1.19-1.44). The authors found no obvious heterogeneity among studies on the association between DII and risk of CVD (P = 0.160; I^2^, = 37.0%). However, there was heterogeneity among studies on the association between DII and mortality from CVD (P < 0.001; I^2^ = 70.8%). In the sensitivity analysis, exclusion of any single study did not materially change the pooled RRs.

Finally, in a systematic review and meta-analysis of 24 studies by Fowler and Akinyemiju (17), individuals in the highest DII categories had a 25% higher overall risk of cancer incidence (RR: 1.25, CI 95%: 1.16-1.35), 75% higher odds of cancer (OR, 1.75; 95% CI, 1.43-2.16), and a 67% higher risk of cancer mortality(RR, 1.67; 95% CI, 1.13-2.48) than the ones in the lowest categories. There was no evidence of heterogeneity between the studies on DII and cancer incidence (I^2^ = 39%, p = 0.083) or cancer mortality (I^2^ = 0.0%, p = 0.548), and there was moderate evidence of heterogeneity between studies that assessed the risk of general cancer (I2 = 48.9%, p = 0.048). After stratifying the results for cancer type, the positive associations remained (RR for breast cancer: RR, 1.12; 95% CI, 1.03-1.22) (RR for colorectal cancer: 1.33, 95% CI, 1.22-1.46) (RR for lung cancer: 1.30, 95% CI, 1.13–1.50). There was no evidence of statistical heterogeneity between the studies that evaluated each type of cancer (breast I^2^ = 0.0%, p = 0.451; colorectal I^2^ = 22.1%, p = 0.277; lung I^2^ = 0.0 %, p = 0.791).

### Limitations of the DII

This study has some limitations. First, we inferred the causality between obesity and inflammation. Many factors besides food components might contribute to inflammation in individuals with obesity (9). Second, sampling biases related to the articles included in this study, such as the use of different tools to analyze food intake and variations in the number of food components analyzed in each study, cannot be denied (14,61). Third, there is a lack of specific dietary questionnaires to evaluate the intake of specific dietary components that can influence inflammation. Finally, it is important to clarify that the current version of the DII is patented (DII^®^) and only the study group that developed it can calculate it. Thus, other investigators cannot explore this tool. However, the patent holders intend to develop applications for computers and smartphones that can be used in patient counseling and dietary interventions in clinical settings (15).

In conclusion, as we have presented, the inflammatory obesity aspect is clear and, although food components seem to play a role in obesity development and inflammatory state, more studies are needed to clarify the relationship between diet and obesity-related inflammation. Data form different studies worldwide in both adolescents and adults demonstrated an association between DII with obesity and cardiometabolic disease. Therefore, DII may be useful in clinical practice for patient advising. Further studies are needed to clarify the pathways and the relationship among the DII, obesity, and cardiometabolic disease.
